# Playfulness and adolescence: mentalization and neuropsychoanalysis perspective

**DOI:** 10.3389/fpsyg.2024.1371747

**Published:** 2024-06-12

**Authors:** Martin V. Kolev

**Affiliations:** Department of Philosophy, Sofia University, Sofia, Bulgaria

**Keywords:** Neuropsychoanalysis, MBT-A, adolescence, mentalization, attachment, psychotherapy, psychodynamic

## Abstract

The article attempts at conceptualizing the basic principles of how adolescents develop, getting out of childhood and proceeding to enter young adulthood. The age period of adolescence is marked with intense emotional states, lines of thinking, beliefs and transitions that caregivers often face challenges making sense of or mirroring. Combining mentalization-based approaches with neuropsychoanalytic findings about how basic emotional systems governing playful behavior work can shed additional light into the communication channels and specificities therapists might consider when engaging in such endeavor.

## Introduction

Adolescence is considered a period of transition in human development, positioned between childhood and adulthood (Zaky, 2016) Often times adults romanticize or demonize it by labeling it “full of hormones,” rebellion, stupidity, the domain of “romantic love” that will fail at a certain point, a crisis that is meant to defy authority, “you have not seen anything yet,” etc. almost forgetting they were once adolescents as well. It is also conceptualized that adolescence holds in itself the onset of the puberty process, i.e., becoming sexually mature alongside other biological, hormonal changes.

Casey et al. (2010) summarized that behavioral changes during adolescence are often seen and described as delinquent without necessarily being against the law. Truly a period that is often times very hard to represent through adequately marked emotional mirroring accompanied by a distinct system of thought on the side of the young person “constituting the essence of the logic of cultured adults and even providing the basis of elementary forms of scientific thought” (Piaget, 1972).

Some authors see adolescence as ending when the individual attains a stable, independent role in society (Taylor et al., 2013). But certainly, if we think about the internal world, there is much more to consider. While moving through that extended natural rite of passage teenagers are almost inevitably experiencing shifts of peer group structure, sexual encounters, first romantic relationships, societal demand, emotional regulation challenges, seemingly independent personal thoughts, never before experienced situations (Guyer et al., 2016). Those same new experiences need to be directed, orchestrated and exercised in a manner allowing proper expansion of choices and corresponding to them methods of satisfying personal and societal needs with a good enough balance between those two aspects. One evolutionary tool mammals and human beings use is playful behavior.

But how do we conceptualize play? What does that lightly sounding word stand for?

According to the Cambridge dictionary, “play” can be defined as spending time doing an enjoyable activity; to take part in a game or other organized activity; to compete against a person or team in a game; to choose a card from the ones you are holding in a card game and put it on the table; to perform an entertainment or a particular character in a play or film; to behave or pretend in a particular way in order to produce a particular effect or result; to help to achieve something.

In science, we have had a long history of how we conceptualize play and its functions, ranging from Schlosberg (1947) who saw play as so insignificant that it’s better to “throw it in the trash bin of science,” developing in a more meaningful way by authors like Baldwin and Baldwin (1977) who listed approximately 30 aspects and functions of play and reaching today’s understanding of play as being not merely an observable or hypothesized behavior but a behavior that is connected to a fundamental and intrinsic neurobehavioral process in the mammalian brain (Siviy and Panksepp, 2011).

We often think of developmental steps mainly as belonging to childhood, or the first years of life. One very interesting idea that connects play and early development is Winnicott’s notion of the transitional object (and transitional phenomena). Winnicott (1953) saw that as a sign of health in the human child that is universal for development.

According to Lavanco (2005), transitional phenomena can also be seen in the developmental task (and potential achievement) for the adolescent to try to think and feel independently, thus constructing a new transitional space, slowly leading to internal changes and in turn to better adaptation to the external world.

In childhood a transitional object might take the form of “something” (toy, blanket) that has the purpose of reducing anxiety and providing self-directed care and comfort in uncertain situations and significant changes, often related to the emotional feeling, resulting from the perception of the absence of the primary caregiver. Transitional objects in childhood seem to have a very different purpose compared to those in adolescence that researchers are not quite sure how to interpret yet – having positive impact or having negative impact on mental health. The purpose of transitional objects in this transitional time (and their meaning) seem to rely even more on the psychological context of the young person. In their study, Erkolahti and Nyström (2009) attempted to clarify the connection between transitional objects in adolescents aged 13 and 14 and depressive symptoms (*N* = 992). What they found is that one third of young people in their study reported having some kind of transitional object. Higher percentage of females had transitional obejcts (37%) than males (18%). This research discussed further the tendency of young people having transitional objects to display more depressive moods but also that prolonged use of a transitional object in adolescence might be linked to vulnerabilities and possible psychopathological processes (Erkolahti and Nyström, 2009). Parallel to that, Bachar pointed out that chronic illness and associated somatic symptoms are felt subjectively by the ill person to diminish and comfort to rise when using transitional objects to self-soothe (Bachar et al., 1998).

Several questions are evoked here: Firstly, if some adolescents need transitional objects to transition better through their transitional period, how much should caregivers interfere? What about therapists? Secondly, if a therapist identifies potentially harmful situations, how should they interfere through safety procedures, interpretations, clinical interventions or techniques and are there common themes among adolescents that we should know about and what to or what not to do with them? And thirdly, is playful behavior the “tool” by which parents and therapists can better engage with adolescents and is it safe to play with emotionally loaded psychological conflicts, interactions and relationships in the family and/or therapeutic encounter?

I would like to argue that Winicott’s idea of finding play in the transitional space can be adapted to neuroscientific findings and produce a hypothesis that play is a system on its own, with its neurological correlates that serves as transitional field into which all other emotional needs are to be exercised and explored with the developmental intention of reaching satisfactory capacity.

In this day and age of “classical-psychoanalytic-theory-destabilizing” neuroscientific findings, we know that human beings inherit the same basic emotional command systems as every other mammal on earth – the field of affective neuroscience managed to formulate seven of them – SEEKING, RAGE, FEAR, LUST, CARE, PANIC/GRIEF and PLAY (Panksepp, 2010). In neuropsychoanalysis, all these basic emotional systems are also seen as drives in their own right. “Drive” here is used both as a reference to Panksepp’s theory and also to Freud’s notion of “Trieb,” that was mistranslated by Strachey as “instinct” (Solms, 2013). Freud (1915) considered that the drive (“Trieb”) “appears to us as a concept on the frontier between the mental and the somatic, as the psychical representative of the stimuli originating from within the organism and reaching the mind, as a measure of the demand made upon the mind for work in consequence of its connection with the body.”

We have all of these emotional drives ready and functioning from birth and it’s our experience with caregivers and environment that “teaches” us how to satisfy them by providing us with predictive models and assumptions about how to face the demands of all seven of them individually and also their interactions and the internal conflicts resulting from them. Predicting the demands of those drives – the felt sensation of a need - is a form of social and inter-subjective learning that is internalized and serves as the basis of our life ahead; accumulated knowledge of this kind is often times meant to solve difficult, “unsolvable” problems while they unfold; what we do in childhood is inventing a way to make the problem “go away.” And it does, at least for a while, until the prediction potentially fails and we feel a certain way dependent on the emotional system that is active at this time. What we feel then is a feeling, a demand from the body upon the mind, that signals us that we need to do some work into the world in order to satisfy our need (Freud, 1895; Solms, 2018).

The way we do this is mainly through trusting our caregivers or trusting the automatic processes of memory functions that allow us to search through our memory storage and find a solution that worked before, at least up until now. But what can be done when predictions fail and we are left with an emotional system that fires out of balance and makes us feel uneasy? (Solms, 2018) We can mainly reflect on what is actually going on; first, bring into awareness that something is off and then divert our attention from “first” and “second person point of view” and attempt at engaging the so called “third person point of view” trying to see “ourselves from the outside and others from the inside” (Allen, 2003).

This useful parallel from mentalization theory suggests the important role of attachment and its connection to social cognition, particularly in children learning about mental states and intentionality at first; when we are little we do not possess the ready skill-set to mentalize but we do come equipped with all the “parts” needed to assemble it through emotionally meaningful interactions with the ones that take care of us (attachment figures) (Fonagy et al., 2002). While developing the capacity to mentalize, the child faces developmental steps that have to do with how minds are seen and interpreted. In one such development [labeled “pretend mode” (Fonagy et al., 2002)], the young mind is able to use the notion of the “other mind” and discriminate it from one’s own but in a specific context which oftentimes happens to be play. In this pre-mentalizing state, thoughts and feelings can be perceived in conscious awareness, discussed, talked about but they do not contribute or correspond to anything real. Once the young mind works through that stage (via growing up, the help of attachment figures, internal integration and consecutive proper mentalizing), learning from experience and the more flexible continuity between feelings, intentions, planning and action can happen in a clearer way for the young person (Midgely et al., 2017).

In this sense care, play, marked mirroring and mentalizing lead to learning (Valle et al., 2016).

With the above mentioned mechanisms in mind we can clearly see how emotional systems and their “pure emotional” firings are to be mentalized in order to make them intelligible, digestible, promoting growth and health. From Panksepp’s seven emotional systems the PLAY drive serves a purpose of directing all other drives to satisfaction; serving a conducting role in a sense, this “conductor” has to be functioning well enough in order for the young person to better manage their needs for curiosity, exploration and change. Furthermore, adolescents carry their childhood predictions about PLAY in puberty but they will inevitably be challenged and changed dramatically in that age period and there is no certainty that those changed predictions will be adequate and working well enough or end in prediction errors (Heffner et al., 2021) that the adolescent will eventually feel as unpleasant sensations, symptoms or recurring patterns of maladaptive behavior or manifest in behaviors of malignant forms of self-care (Townsend et al., 2022). As we can see, adolescents are carrying a lot on their shoulders and this weight can often burden them. Some of it can be “mentalizable” or a subject of play by benevolent adults (e.g., parents, teachers, therapists) but other parts of it are better left untouched as they are the domains of sensitive topics like emerging sexuality, romantic relationships, friendship and betrayal, substances, eating habits, mental health, perspectives on the future, etc. (Rodriguez, 2018).

With sensitive topics, such as the ones listed here, a crucial factor sometimes is uncertainty and confusion about mental states that are known to mediate anxiety. (Martin-Gagnon et al., 2023) In parallel, anxiety is known for fueling chronic conflicts in family relationships (Carr, 2023). This way anxious feelings from the adolescent - that is struggling to make sense of what is going on in their world - can be picked up by family members and either be metabolized by them and mirrored back in a digestible form or disrupt mentalizing and create conflicts if not mentalized well enough.

Furthermore, we know that playful behavior and curiosity seem to promote mentalizing and good mentalizing seems to promote symbolic play in return (Halfon and Bulut, 2017).

We think that mentalizing the emotional components of adolescent functioning and not their sensitive developmental tasks *per se* proves to be a crucial point of intersubjective meaning-making between the young person and their families or in therapy. Sensitive topics such as emerging sexuality can evoke strong feelings of shame or disgust that can lead to disruption of mentalizing and heightened levels of anxiety or alienation of the adolescent. Adding the concept of PLAY as an emotional system that tends to govern other emotional needs can be beneficial when applying mentalization-based approaches to helping adolescents transition better through their path to young adulthood.

In accordance with Barnett (2005), we think that to be able to reach the adolescent emotionally through play we also have to focus on and understand the internal qualities of the individual that make play possible, i.e., to be playful.

So, how playful should we be in our therapeutic interaction with adolescents? It is our view that it depends on how the adolescent you are trying to be playful with defines and sees play, when does that happen and who is the particular adolescent in question. Regarding feelings associated with the intrinsic need to play, our automatic implicit predictions inevitably navigate our attention and responses, and with adolescents the ability to mentalize can be overshadowed by those same feelings, especially when they are “too hot.” In order to help them achieve better emotional regulation, it seems we need to be careful around their sensitive topics and conflicting predictions, try to be playful in our interventions, but closely monitor if something that we perceive as play is not taken too literally and experienced as traumatic by the young person.

## Data availability statement

The raw data supporting the conclusions of this article will be made available by the authors, without undue reservation.

## Author contributions

MK: Writing – original draft.
